# The metacognitive experience of time passing in Chinese college students: scale development, structure verification, and influencing factors

**DOI:** 10.3389/fpsyg.2023.1180863

**Published:** 2023-06-28

**Authors:** Xide Yu, Jiafan Liu, Yaohui Lin, Xianli Chen, Cheng Lu

**Affiliations:** ^1^Department of Applied Psychology, School of Education Science, Guangdong Polytechnic Normal University, Guangzhou, Guangdong, China; ^2^School of Medicine, Shaoguan University, Shaoguan, Guangdong, China; ^3^Student Affairs Office, Guangzhou South College, Guangzhou, Guangdong, China

**Keywords:** metacognitive experience, experience of time passing, ruminative experience, emotional experience, reliability and validity

## Abstract

The experience of time passing (ETP) is also the consciousness of the progress of life. ETP contributes to time regulation and life management, which basically conforms to the metacognitive theory. Also, the traditional Chinese cultural approach to time emphasizes ETP. It is an indispensable part of Chinese education and culture to strengthen one’s appreciation of time by emphasizing the passage of time. In combination with the above two points, ETP equals metacognitive experience of time passing (METP) to a certain extent. However, we currently know little about the connotations of METP. To better understand traditional Chinese time culture, and referring to the concept of metacognition and model of time experience as proposed by Western scholars, the current study combined the results of open and semi-structured interviews, to explore the structure of METP in Chinese college students and developed a questionnaire with which to measure it. Using convenience sampling, 2,876 college students were recruited, the interview, and the reliability and validity tests were carried out. Five hundred and seventy-nine college students were tested a second time to investigate the correlation validity between METP and Ruminative Responses, time attitude, and meaning in life. The results led to the development of the METP Scale which contains 15 items and assesses two factors: ruminative and emotional experience of time passing. The two-factor model was well fitted, and invariable in measurements across gender, grade, and major. The internal consistency coefficients of the scale and its two factors ranged from 0.82 to 0.89, the half-point reliability between 0.76 and 0.88, and the retest reliability ranged from 0.77 to 0.78. METP Scale has good correlation validity, meanwhile, the results of regression analysis showed that symptom rumination, positive past, negative present, positive future, and searching for meaning in life significantly predict the intensity of METP.

## Introduction

Time and its passage is a subject of study both ancient and modern. The discussion of the passage of time depends on the understanding that time exists. However, does the world we live in exist in time at all? This question has been long debated (Yehezkel, 2013; Liu et al., 2015). Physicists from even earlier, such as Isaac Newton, believed that time was absolute, never lingering nor standing still (Rynasiewicz, 1995; Gefter, 2008; Thomas, 2022), and has nothing to do with humans (Dieks, 1988; Gao, 2001). Other physicists, such as Albert Einstein, argued that time and space are inseparable entities, that time is an important property of space and that it is an illusion that we see space from different perspectives (Li, 1995; Baranchuk, 2019). Overall, philosophers’ attitudes towards time and its passage are relatively contradictory and complex, with some believing that absolute time may not exist, but rather that it is a kind of consciousness experience in which time and its passage do exist (Liu et al., 2015; Riggs, 2017; Montemayor and Wittmann, 2022). Another group of philosophers, through logical deduction, have proven that time and its passage do not exist, explaining that time and its passage do not have a “reality,” it can only be called an “experience,” and experience is contingent, that it can be experienced and also cannot be experienced, therefore, the experience of time and its passage do not have the attribute of absolute existence (Prosser, 2007; Power, 2009; Yehezkel, 2013).

From the perspectives of physics and philosophy, it is less likely to make a definitive conclusion about the existence of time and its passage, and the related debate seems to be continued. However, a very real and thought-provoking question is: If time does not exist (or barely exists), then why do we have such a strong sense of it? Why do we often feel like time is passing so quickly or slowly? Obviously, time and its passage exist in our brain and consciousness. It is noted that a large number of empirical studies have explored time perception and self-awareness within the context of psychology, and proved that time perception is closely related to such cognitive functions as attention, working memory, and thinking (Droit-Volet, 2013; Matthews and Meck, 2016; Droit-Volet et al., 2018; Nazari et al., 2018). Prior psychological studies have also found that the distortion of time processing can be related to emotions such as social exclusion (Twenge et al., 2003), sadness (Gil and Droit-Volet, 2009), and anxiety (Liu and Li, 2019). Several scholars even suggested that temporal experience is an exclusive feature that can be used to judge the incidence and severity of depression (Ratcliffe, 2012; Yoshiike et al., 2022). Altogether, these findings confirm the link between time perception and self-awareness.

Recognizing the importance of understanding time perception and its practical significance, and considering that a sense of time passing is also an important part of time perception, some scholars have also proposed that the sense of time passing is a typical time consciousness (Wittmann et al., 2015; Martinelli and Droit-Volet, 2022), making it clear that research on the sense of time passing is also interesting and essential. However, there is relatively little current psychological research on time passing (Yu et al., 2018a). Unlike the estimation of a length of time (done mostly on the millisecond, second, and minute scales), the sense of time passing is more concerned with a long span of time or a lifetime, and how one experiences that lifetime passing. Western scholars have generally defined the sense of time passing as an individual’s cognitive judgment of how fast or slow time passes (Droit-Volet and Wearden, 2016; Di Crosta and La Malva, 2017; Droit-Volet et al., 2017). While, some scholars believe that judging the speed of time passing is a relatively general and superficial experience, which cannot fully contain individual feelings and thoughts about time passing (Wearden, 2015; Yu et al., 2017). Therefore, scholars have recently expanded or rephrased the concept from the sense of time passing to the experience of time passing (ETP) which combined the connotation of sensation, perception, feeling, and consciousness of time passing (Droit-Volet, 2019; Droit-Volet and Dambrun, 2019; Martinelli and Droit-Volet, 2022). However, there is no clear theoretical explanation of content and structure of the experience of passage of long time or lifetime. Furthermore, there are also few researches on the connotation and structure of ETP from the perspective of psychometrics. In view of this, this study aims to explore the concept of ETP by means of psychological measurement, in order to enrich the psychological theory and practical implication of ETP.

## Literature review

### The S-shape model

**Figure 1:**
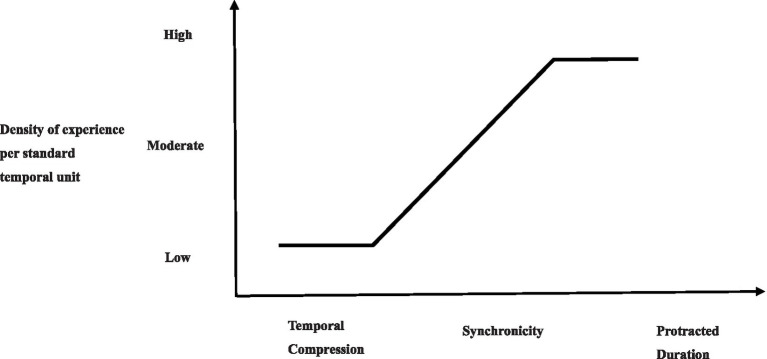
The relationship between time perception and density of experience per standard temporal unit. Reproduced from Flaherty (2018) with permission from Cambridge University Press. License number: 5574730388916.

The S-shape model was proposed by Flaherty (2018) as an earlier theoretical model that discussed the velocity of time passing. However, it should be clarified that Flaherty (2018) did not explicitly express the content and structure of the ETP. Instead, he focused on three states (or results) of ETP: temporal compression, synchronicity, and protracted duration. In the S-shape model, changes in the ETP occur in the context of standard units of objective time. In some cases, we feel that time passes quickly or slowly. In other cases, our subjective experience of time synchronizes roughly with objective time. The S-shape model can be used to explain the full range of the temporal experience, as shown in Figure 1.

The S-shape model explains in detail the reasons for the three kinds of results of the ETP, that is, change in the ETP reflects the change in experiential density (ED) of each standard unit of time. In turn, the ED of each standard unit of time is mainly based on the dynamics of social interaction. Interestingly, Flaherty had a unique interpretation of ED, and argued that we cannot take for granted that ED is the absolute number of situations, events, or even human interactions; instead, ED should be regarded as a cognitive involvement brought about by these situations, events, and interpersonal interactions. It is quite possible that something has happened many times over, but that our cognition remains blank, causing us to feel that time is passing quickly and resulting in a phenomenon of time compression. Meanwhile, other events, even though they happen only once, may create a strong enough impression to create a strong density of experience that ultimately gives us a sense of passing slowly or having a protracted duration (Flaherty and Meer, 1994; Flaherty et al., 2005; Flaherty, 2018). Indeed, findings have shown that cognitive novelty, complexity, and significance of situations and events are effective predictors of ETP (Larson and von Eye, 2006). In light of this, we can deduce that thoughts associated with the passage of time may be an important part of the ETP.

### Metacognition and metacognition of time passing

Metacognition is a concept proposed by Flavell, an American psychologist. The main feature of metacognition is that it connects perception, memory, thinking, and language, which until that point had been traditionally considered to be separate from one another. Flavell emphasized the integration of these mental processes, which then act collectively on our cognitive system to create a superior perspective (Flavell, 1976, 1979, 1981). In short, metacognition can be defined as, “cognition, originally a cognitive process, taken as a cognitive object, that is, the cognition of cognition” (Wang and Guo, 2000). Flavell (1979) believed that metacognition mainly includes two important elements: metacognitive knowledge (MK) and metacognitive experience (ME). Meanwhile, Chinese scholars Wang and Guo (2000) also agree with Flavell (1979) classification on metacognition. Specifically, MK refers to fragments of knowledge that are not only related to the cognitive subject but also to various tasks, goals, activities, and experiences (Flavell, 1979; Wang and Guo, 2000). In contrast, ME is a cognitive or emotional experience that accompanies and is subordinate to intellectual activity. MK, as a necessary support system for cognitive activity, provides an empirical background for cognitive regulation (Flavell, 1979; Wang and Guo, 2000). Meanwhile, ME provides psychological motivation for cognitive regulation, and can awaken Meta-cognitive knowledge reserved in the memory system. Importantly, ME has been shown to play a role between metacognitive knowledge and metacognitive monitoring or regulation (Wang and Guo, 2000).

According to Flavell (1979)’s elaboration of metacognition, we can conceptually define metacognition on time passing. Metacognitive knowledge of time passing (MKTP) refers to an individual’s cognition of the factors that affect the process and results of their perception of time passing. After several cognitive activities, an individual will gradually accumulate knowledge about key factors and ways that they affect their perception of time passing, which is defined as the MKTP. Metacognitive experience of time passing (METP) refers to an individual’s awareness and understanding of a situation related to their sense of time passing, including both metacognitive emotional and thinking experiences of time passing. Lamotte et al. (2014) was the first to research the metacognition of time passing, investigating individuals’ understanding of the factors influencing their awareness of time passing using a questionnaire. In their first study, 532 young adults were asked to answer an initial questionnaire of 106 questions related to a number of different factors (e.g., psychostimulants, body temperature, age, attention) which were known to affect people’s awareness of the passage of time. The results of exploratory factor analysis showed that two influencing factors could be extracted: attention and emotion. In their second study, they verified the two-factor structure with data from 212 college students (Lamotte et al., 2014). Following Flavell’s concept of metacognition, the research content of Lamotte et al. (2014)‘s study could be defined as the metacognitive knowledge of time passing (MKTP). In contrast, few empirical studies currently exist on METP, as the other metacognitive factor of time passing. Therefore, the present study chose to investigate METP.

### The ETP in Chinese time culture

Although there are few studies on METP in western populations, Chinese scholars have long discussed the emotional and cognitive experience of the feeling of the passage of time. For example, when people are aware of time passing, they generate feelings of pain, anxiety, panic, and guilt (Yu et al., 2017, 2018a,b, 2021; Liang, 2021), and they will adjust or change their thoughts and ideas (Yu et al., 2017, 2018a,b), both of which are, to some extent, preliminary explorations of METP. However, for the following two reasons, these studies cannot be completely equated with METP. First, the abovementioned studies specifically explored the feeling of the passage of time, which did not rise to the level of metacognition. Second, the feeling of the passage of time as studied by Yu et al. (2017). also assessed a dimension of cognitive susceptibility, which cannot be well incorporated into the conceptual connotation of METP (but can be integrated into MKTP). Therefore, the structure and content of METP still need further exploration, both theoretically and empirically.

Two points should be considered when discussing METP in the context of Chinese time culture. First, previous theoretical and empirical explorations of ETP in western populations have been based on standard time units of the technical time system, that is, the second, minute, and hour as shown on a clock, or the day, month, and year on a calendar. In other words, they have explored how fast or slow time passes, or whether time compression, synchronicity, or time extension exists according to the density of the experience as measured in standard units (Flaherty and Meer, 1994; Flaherty, 2018). Clock time is the product of western industrialization, and is a technical sense time, with characteristics that are typically linear and quantitative, as is more popular in Western culture. Qualitative time exists in contrast to technical time which has cyclic characteristics, and is typically measured according to events such as a meal, a cup of tea, or even a pillar of incense in Chinese expression (Yu, 2022). Qualitative time is more accepted in eastern cultures, especially in China, where people have relied on qualitative or event time systems for thousands of years before the advent of technological time systems. Certainly, with the industrialization and modernization of China, both technical and qualitative time systems began to occupy equal proportions of the daily life of Chinese people (Yu, 2022). This raises an interesting question of cross-cultural differences and integration, however. For contemporary Chinese people, especially for college students who have been more exposed to Western culture, are the connotations and extensions of METP consistent with research findings based on the Western technological time system? Second, traditional Chinese time culture not only emphasizes the cyclicity of time, but also emphasizes the background context when individuals form a concept of time. That is, when the Chinese talk about time, they do not just refer to time itself but to a mental image that integrates objects such as time, space, substances, people, and events (Su and Ye, 2020; Yu, 2022). This provides an entry into exploring the connotations and structure of Chinese people’s METP.

## Related concepts or potential influencing factors

### Time perspective or time attitude

Time perspective refers to the sum of the cognitive concepts held by individuals regarding the past, present, and future (Zimbardo and Boyd, 1999), which includes Past-Negative, Present-Hedonistic, Future, Past-Positive, and Present-Fatalistic. One study using an online survey of 423 participants aged 17 to 81 in a Western context examined the relationship between time perspective and subjective speed of time passing in everyday life (Wittmann et al., 2015). The results showed that the present hedonistic perspective was related to a faster subjective experience of the passage of the previous week; the future perspective was associated with a generally faster subjective experience of the passage of time overall; in contrast, a balanced time perspective was positively correlated with a slower subjective experience of the passage of the previous 10 years (Wittmann et al., 2015). Meanwhile, the results of a survey of 928 Chinese college students also showed that having a Past-Negative, Present-Hedonistic, or Future time perspective positively predicted one’s overall FPT (feeling of the passage of time) score (Yu et al., 2018b). While considering these two studies’ results, it should be noted that there was a large age difference between the two participant groups in these two studies. Furthermore, the time perspective scale used in both studies lacked a detailed division of future, that is, specifying future positive and future negative, which is crucial for adolescents which did not rise to the level of metacognition in the stage of identity development. Hence, at least for the college students, the relationship between ETP and time perspective requires further exploration. Mello and Worrell (2007) found that the Zimbardo Time Perspective Inventory showed poor reliability and validity in adolescents, and in response, developed the Adolescent Time Attitude Inventory (ATAI). ATAI has relatively good reliability and validity indeed among Chinese teenagers (Li et al., 2021; Li and Lv, 2022). Focusing on adolescents’ attitudes or emotional experiences regarding their past, present, and future (Mello and Worrell, 2007; Mello and Worrell, 2015), this measure provides a standardized tool to explore factors influencing METP in Chinese college students.

### Rumination response

Rumination response (RR) is a way of thinking that repeatedly focuses on negative emotions and corresponding events. A more specific operational definition is that it is when individuals focus their attention on their own depressive symptoms and potential causes of their thoughts and behaviors (Nolen-Hoeksema and Morrow, 1991; Han and Yang, 2009). Rumination is a typical metacognitive representation by definition. Previous studies have shown that FPT (feeling of the passage of time) contains both introspective and secondary negative emotions (i.e., guilt, pain, regret, and panic; Yu et al., 2017), and individuals have been shown to have a tendency to construct a negative past self (Zhang et al., 2021). There is also a correlation between depression and other negative emotions and time experience (Ratcliffe, 2012; Yu et al., 2018a). Thus, we speculated that rumination, as a typical thinking experience, is one of the potential influencing factors or effective predictors of METP.

### Meaning in life

Meaning in life (MIL) refers to the degree to which one grasps, understands, or sees the meaning of their life, accompanied by the degree to which they are aware of their own life purpose, mission, and primary goal. This includes the presence of meaning as well as the search for meaning (Steger, 2009; Wang, 2013). The focus of METP is a lifetime or lived time, and existing studies have shown that there is a significant positive correlation between MIL, time value, and time management (Dong and Yin, 2021; Chen and Wang, 2022). Furthermore, according to the S-shape model, experience density affects experience of time passing. Although we cannot completely equate experience density with meaning in life, the view that the acquisition of MIL is based on experience is accepted in academic circles (Zhao et al., 2017). Based on this, we speculated that MIL will also be one of the factors influencing METP.

## The present study

Although we can theoretically demonstrate the rationality of the concept of METP, and there are currently some standardized scales that examine the consciousness of time passing from a variety of aspects, there are two reasons that drove us to carry out a new exploration of METP. First, there is no standardized scale directly focused on METP, which means that even if a concept structure is determined to be valid, it still lacks empirical testing. Second, neither the existing Metacognitive Scale of Time Passing (Lamotte et al., 2014) nor the Feeling of Passage of Time Scale (Yu et al., 2017) fully and effectively cover the concept connotation of the metacognitive experience as proposed in this study. Specifically, the MSTP belongs more to MKTP (metacognitive knowledge of time passing), conceptually, but lacks a metacognitive experience perspective. Meanwhile, although the FPTS contains elements of the metacognitive experience (i.e., emotional experience), the dimension of metacognitive thinking experience of time passing is not clear. Therefore, the concept and the standardized measuring tool of METP requires further exploration.

College students are in the middle and late stages of their identity development, with a great deal of plasticity in their thinking about time and life (Yu et al., 2021) and their meaning of existence (Shek et al., 2021), as well as in their emotional regulation abilities (Silvers, 2022). The METP, as an intermediary element of the metacognitive activity of time passing, can activate the consciousness of time passing, regulate the monitoring of time passing, and thereby strengthen the perceived value of time and time management ability. In addition, as emotions, degree of arousal, and thinking are closely related to time perception (Liu and Li, 2019), it is also of potential clinical benefit to deduce emotional states and thinking characteristics from the perspective of METP, especially for some psychological symptoms that are highly latent and difficult to determine and evaluate, such as suicidal ideation, latent depression, or implicit social rejection.

Importantly, against the background of the combination of Chinese event-based qualitative time culture and Western technological time culture, the current study explores the universality and uniqueness of the conceptual connotation and denotation of Chinese college students’ METP. What is it? How can we reasonably and effectively evaluate METP? And, in view of the synchronous emphasis of time, space, people, and events in traditional Chinese time culture, apart from the background indicators of time experience, what other influencing factors affect Chinese college students’ METP?

## Methods

### Respondents

Convenience sampling was utilized to recruit college students from across four universities in Guangdong Province, China. Sample 1 took part in an open-ended, semi-structured interview, while the remaining four samples were tested using electronic questionnaires in class. All the recruited students are non-English majors. In addition, according to the basic principles of psychometrics, at least five samples are required to complete the interview, exploratory factor analysis of the initial questionnaire, confirmatory factor analysis, correlation validity test and retest reliability test, respectively. For sample details, see Table 1.

**Table 1:** Information of the total sample size of this study.

Sample number	Aims	Total number	Gender		Grade				Age range (M ± SD)
			Male	Female	Freshman	Sophomore	Junior	Senior	
Sample 1	Open-ended and semi-structured interviews	8	4 (50%)	4 (50%)	2 (25%)	2 (25%)	2 (25%)	2 (25%)	17–23 (19.88 ± 2.03)
Sample 2	Items analysis and exploratory factor analysis	1,033	309 (29.90%)	724 (70.10%)	416 (40.30%)	373 (36.10%)	151 (14.6%)	93 (9.0%)	16–23 (19.29 ± 1.39)
Sample 3	Confirmatory factor analysis, internal consistency reliability analysis, split-half reliability analysis	1712	488 (28.5%)	1,224 (71.5%)	436 (25.5%)	1,050 (61.3%)	166 (9.7%)	60 (3.5%)	17–27 (20.25 ± 1.33)
Sample 4	Criterion correlation validity analysis, regression analysis of influencing factors	579	143 (24.7%)	436 (75.3%)	218 (37.7%)	300 (51.8%)	34 (5.9%)	27 (4.7%)	16–27 (19.01 ± 1.38)
Sample 5	Retest reliability	123	18 (14.6%)	105 (85.4%)	0	6 (4.9%)	79 (64.2%)	38 (30.9%)	19–24 (20.80 ± 0.89)

### Procedure

This study was approved by the Ethics Committee of the School of Education Science of Guangdong Polytechnic Normal University. The study procedures followed the American Psychological Association ethics code, which included confirming participants’ informed consent, a confidentiality agreement, and a statement of anonymity. The measurement was conducted online, and all participants were informed of the process they would follow before taking part in the study, and it was stressed that their participation was voluntary and that their data would be kept confidential. The informed consent was included as a document at the beginning of the questionnaire, and participants received the following instructions: “Before completing the questionnaire, please read the informed consent of this study carefully. Receipt of your data in the questionnaire system means that you have read the informed consent and agreed to its contents. If you do not wish to take part, you can exit immediately.”

After the preparatory work was completed, the specific procedure of the research was as follows.

Sample 1 took part in an open and semi-structured interview. The participants were recruited through advertising. Before the interview officially starts, the participants will be informed that the general content of the interview will be recorded, and the interview content will also be anonymous and confidential. If the participants agree, the interview will start, if they do not agree, they can leave the psychological counseling room immediately. During the interview, the participants were asked several questions, such as, Do you experience the passage of time? How would you experience the passage of time? Do you experience the passage of time actively or passively? Interviews were conducted one-on-one in the psychological counseling room. Overall, Sample 1’s participants acknowledged the existence of ETP and described ETP as a manifestation of intensity, thought, and emotion of the feeling of the passage of time.

Next, based on the results of the semi-structured interview, the initial questionnaire was developed with consideration of the perspective of metacognitive thinking and emotional experience. Sample 2 was tested using the initial METP questionnaire, and exploratory factor analysis (EFA) was performed.

Third, METP questionnaire after EFA was used to test sample 3, and confirmatory factor analysis (CFA) was conducted.

Fourth, sample 4 was tested by using METP scale, Time Attitude Inventory (TAI), Rumination Response Scale (RRS) and Meaning in Life Scale (MILS), to test the validity of the criterion and explore the influential factors of METP.

Fifth, the METP scale was used for repeated measurements of sample 5 at a four-week interval to test the retest reliability.

### Measurements

#### The metacognitive experience of time passing scale

This scale was developed through the course of this study, and the first version of it consisted of 24 questions and was scored on a five-point scale ranging from 1 (strongly disagree) to 5 (strongly agree). The final version of the METP scale contains 15 items, its structure, and specific items are shown in Table 2.

**Table 2:** Results of item-total statistics.

Item number	Scale average if item deleting	Scale variance if item deleting	Corrected item-total correlation	Alpha if item deleted	Item difficulty
N1	69.95	90.850	0.441	0.877	0.727
N2	70.12	89.573	0.523	0.875	0.692
N3	70.01	89.014	0.561	0.873	0.714
N4	70.06	89.399	0.555	0.874	0.704
N5	69.94	90.84	0.429	0.877	0.728
N6	70.17	88.924	0.566	0.873	0.682
N7	70.37	90.025	0.508	0.875	0.642
N8	70.21	89.640	0.515	0.875	0.675
N9	69.98	89.405	0.560	0.874	0.721
N10	69.98	89.217	0.574	0.873	0.722
N11	70.19	89.987	0.491	0.875	0.679
N12	70.38	91.638	0.334	0.880	0.641
N13	70.26	88.437	0.563	0.873	0.666
N14	70.52	90.48	0.416	0.878	0.612
N15	70.49	89.479	0.452	0.877	0.618
N16	70.41	89.151	0.514	0.875	0.635
N17	70.58	90.277	0.443	0.877	0.601
N20	70.21	91.250	0.372	0.879	0.674
N21	70.57	91.838	0.305	0.882	0.603
N22	70.11	90.752	0.423	0.877	0.694
N23	70.55	91.233	0.374	0.879	0.607
N24	70.19	89.623	0.510	0.847	0.679

The number of items is 22. Two items with item - total score correlation lower than 0.4, N18 and N19, were deleted before item analysis.

#### Time attitude scale

This scale was developed by Worrell et al. (2011), and translated into Chinese by Li et al. (2021), to measure individuals’ attitudes toward the past, present, and future. The Chinese version of the questionnaire contains 30 questions measuring six dimensions: positive past (PPA), negative past (NPA), positive present (PPR), negative present (NPR), positive future (PFU), and negative future (NFU), with five items for each dimension. Each item is rated on a five-point scale ranging from 1 (strongly disagree) to 5 (strongly agree). In this study, the internal consistency *α* coefficient for the total scale and the six dimensions were 0.70 (TAS), 0.84 (PPA), 0.85 (NPA), 0.88 (PPR), 0.86 (NPR), 0.87 (PFU), and 0.73 (NFU), respectively. The six dimensions of the TAS also showed good reliability in surveys with Chinese college students as participants, with internal consistency coefficient *α* reaching 0.62 ~ 0.83 (Li and Lv, 2022) and 0.77 ~ 0.86 (Li et al., 2021), respectively.

#### Rumination scale

This scale was developed by Nolen-Hoeksema and Morrow (1991), and the Chinese version was translated by Han and Yang (2009), to measure the degree of spontaneous rumination one experiences during stressful events and situations. There are 22 items in total, each of which is rated on a four-point scale ranging from 1 (never) to 4 (always). This scale measures three dimensions, symptom rumination (SRU, 12 items), brooding (BRD, 5 items), and reflective pondering (RPD, 5 items). In this study, the internal consistency coefficient *α* for the full scale and the three dimensions were 0.95 (RS), 0.92 (SRU), 0.82 (BRD), 0.76 (RPD), respectively. This scale showed high reliability in previous study, and the internal consistency coefficient *α* also reached 0.95 (Ye et al., 2020).

#### Meaning in life scale

This scale was developed by Steger et al. (2006) and revised by Wang (2013) to test one’s degree of understanding of the meaning in life (MIL), accompanied by the degree of awareness of one’s own life purpose and mission. A total of 10 items are used to measure two dimensions of MIL, the presence of meaning (MLQ-P, 5 items), and the search for meaning (MLQ-S, 5 items). Each item is rated on a seven-point scale ranging from 1 (completely inconsistent) to 7 (completely consistent). In this study, the internal consistency coefficient *α* of the total scale and the two dimensions were 0.88 (MIL), 0.90 (MLQ-S), and 0.82 (MLQ-P), respectively. This scale also has high reliability in past research, and internal consistency coefficient *α* of the total scale reached 0.828 (Yu and Du, 2023).

### Statistical analysis

SPSS 26.0 and Mplus 8.30 were used to analyze the data, including descriptive statistics, correlation analysis, EFA, CFA, reliability test, and regression analysis.

## Results

### Selection of items

#### Analysis of sample 1 questionnaire data

First, in consideration of the distribution of the total score, the bottom 27% and the top 27% were categorized as the high and low score groups, respectively. The results of the independent sample *t*-test showed that there were significant differences in the scores of the two groups on the 24 initial items (*t* = −8.13 ~ −22.54, *p* < 0.001). Second, the correlation coefficient between each item and the total score was then calculated. Two items whose correlation coefficient between the item score and the total score was less than 0.4 were deleted; the correlations between the remaining 22 items and the total score was 0.43 ~ 0.61 (*p* < 0.001). Third, we did item difficulty analysis on the remaining 22 items, and the results showed that the difficulties ranged from 0.601 to 0.728 (for details, see Table 2), all within the standard range of 0.2 ~ 0.8 proposed by scholar earlier (Wu, 2008). Finally, we tested to see whether the Cronbach *α* coefficient of the scale would be significantly improved after the deletion of each individual item. The results showed that the original Cronbach’s *α* coefficient of the questionnaire was 0.881, and the reliability of the questionnaire remained between 0.873 and 0.882 after each deletion of an item. Of all the items, the deletion of item 21 caused the reliability of the questionnaire to reach 0.882. Therefore, item 21 was deleted from the scale. For details, see Table 2.

#### Exploratory factor analysis

EFA was carried out for the remaining 21 items using the maximum orthogonal rotation of principal component variance (KMO = 0.91, χ^2^ = 7192.41, *df* = 210, *p* < 0.001). According to the principles of having a factor loading greater than 0.40, commonality greater than 0.30, the CITC (corrected item-total correlation) value is not less than 0.4 (for details, see Table 2), and the logical association between items, a total of six items were deleted, after which two factors with eigenvalues greater than one were extracted, leaving a total of 15 items, which could explain 49.39% of the total variance. Factor 1 contained 10 items which involved respondents’ experience of rumination in the perceiving passage of time, and thus was named ruminative experience of time passing (RETP). Factor 2 contained five items which were related to respondents’ emotional experience during the perception of time passing, and was thus named emotional experience of time passing (EETP). See Table 3 for details.

**Table 3:** Exploratory factor analysis of college students’ METP (*n* = 1,033).

Item #	Items	Factor 1	Factor 2	Commonality
		Loading	Loading	
N5	Eng: The sense of time passing makes me appreciate the present moment more. Chi: 时间流逝感会让我更加珍惜当下。	**0.72**	−0.14	0.53
N3	Eng: The sense of time passing makes me reflect on myself. Chi: 时间流逝感会促使我反思自我。	**0.71**	0.13	0.52
N10	Eng: The sense of time passing forces me to think more about the past, present, and future. Chi:时间流逝感促使我更多地思考过去、现在与未来。	**0.70**	0.18	0.52
N9	Eng: The sense of time passing reminds me that I need to think about changing things in my life. Chi: 时间流逝感提醒我应该要考虑调整生活状态了。	**0.69**	0.18	0.51
N6	Eng: The sense of time passing makes me think about my relationship to my surroundings. Chi: 时间流逝感会促使我思考与周边的关系。	**0.66**	0.18	0.47
N2	Eng: The sense of time passing prompts me to look inside myself. Chi: 时间流逝感会促使我内观自我。	**0.63**	0.18	0.43
N1	Eng: The sense of time passing reminds me to do things. Chi: 时间流逝感在提醒我要做点什么。	**0.62**	−0.05	0.39
N4	Eng: The sense of time passing forces me to face my true self. Chi: 时间流逝感促使我面对真实的自己。	**0.61**	0.24	0.43
N8	Eng: The sense of time passing makes me think about my relationships with others. Chi: 时间流逝感促使我思考自己与他人的关系。	**0.55**	0.27	0.38
N7	Eng: The sense of time passing makes me focus more on details. Chi: 时间流逝感会让我更加聚焦细节。	**0.49**	0.28	0.32
N15	Eng: The awareness of time passing makes me panic. Chi: 意识到时间流逝会让我惊慌失措。	0.06	**0.82**	0.67
N14	Eng: The awareness of time passing makes me feel pain. Chi: 意识到时间流逝会让我感到痛苦。	0.05	**0.78**	0.61
N16	Eng: The awareness of time passing makes me feel regret. Chi: 意识到时间流逝会让我感到懊恼。	0.17	**0.77**	0.63
N17	Eng: The awareness of time passing makes me feel more emotional. Chi: 意识到时间流逝会让我情绪波动较大。	0.13	**0.69**	0.49
N13	Eng: The awareness of time passing makes me feel guilty. Chi: 意识到时间流逝会让我感到内疚。	0.35	**0.63**	0.53
	Eigenvalue	4.28	3.13	
	Explanation rate of variation (%)	28.50%	20.89	

Eng = English, Chi = Chinese. The bold and underlined formatting means the loadings that after converging to a factor through rotation.

### Validity analysis

#### Validity of structure

CFA was carried out using the data from Samples 3 and 4 to verify the structural validity of the METP Scale. The results showed that the two-factor model fit the data well, and all indicators met the criteria. We combined the two-factor model into a single-factor model for verification, and the results showed that the single-factor model was not as good as the two-factor model. The specific fitting indices of each model are shown in Table 4.

**Table 4:** Comparison of confirmatory factor analysis results.

Sample	Model	AIC	BIC	*a*BIC	χ^2^	*df*	χ^2^/*df*	RMSEA	CFI	TLI	SRMR
Sample 3	Two-factor	**49611.247**	**49861.736**	**49715.600**	**289.581** ^***^	**89**	**3.25**	**0.036**	**0.963**	**0.956**	**0.033**
Sample 3	Single factor	51176.051	51421.095	51278.135	1175.764^***^	90	13.06	0.084	0.799	0.756	0.083
Sample 4	Two-factor	**16755.259**	**16955.879**	**16809.847**	**319.416** ^***^	**89**	**3.59**	**0.067**	**0.904**	**0.887**	**0.064**
Sample 4	Single factor	17699.259	17895.518	17752.660	914.823^***^	90	10.16	0.126	0.656	0.599	0.112

^***^p < 0.001. The bolded values represent the fit indexes of the optimal two-factor model. AIC, Akaike; BIC, Bayesian; aBIC, sample-size adjusted BIC; *χ*^2^, Chi-square value; df, degree freedom; RMSEA, root-mean-square error of approximation; CFI, comparative fit index; TLI, Tucker-Lewis index; SRMR, standardized root-mean-square residual.

#### Measurement invariance

MI is to test the equality of the relationship between observed variables and latent variables among different samples after the structure of latent variables is confirmed (Guo et al., 2022). It is an important index reflecting the stability and test quality of measurement tools across populations (Cheung and Rensvold, 2002). For the METP scale developed in this study, we also need to verify its structural stability in different subcategories of the same population (college students). Notably, recent research has shown significant differences in the feeling of the passage of time (FPT) across a number of demographic variables. For example, female college students’ FPT was significantly stronger than that of male college students; FPT of students majoring in humanities and social sciences is significantly higher than that of students majoring in natural science and technology. Meanwhile, college students’ FPT increases with grade (Yu et al., 2022). In context of these findings, we chose to examine the measurement invariance (i.e., structural stability) of METP scale on three variables: gender, grade, and major. Using the data from Sample 3, we carried out four kinds of MI tests, configural, metric, scalar, and strict MI. As shown in Table 5, the results supported the configural invariance of the two-factor structure of the METP Scale in terms of gender, grade, and major. Meanwhile, metric, scalar, and strict MI were also supported, and the variations of CFI and RMSEA did not exceed the suggested critical values, that is, ΔCFI ≤0.01 and ΔRMSEA ≤0.015 (Cheung and Rensvold, 2002).

**Table 5:** Results of measurement invariance test.

Model	*χ* ^2^	*df*	CFI	RMSEA	MC	ΔCFI	ΔRMSEA
**Gender**							
M_0_ (configural)	655.517	178	0.951	0.056			
M_1_ (metric)	668.574	191	0.951	0.054	M_0_ vs. M_1_	0.000	0.002
M_2_ (scalar)	706.623	204	0.948	0.054	M_1_ vs. M_2_	0.003	0.000
M_3_ (strict)	843.604	219	0.935	0.064	M_2_ vs. M_3_	0.013	−0.01
**Grade**							
M_0_ (configural)	1011.891	356	0.934	0.066			
M_1_ (metric)	1056.443	395	0.933	0.063	M_0_ vs. M_1_	0.001	0.003
M_2_ (scalar)	1107.901	434	0.932	0.060	M_1_ vs. M_2_	0.001	0.003
M_3_ (strict)	1273.080	479	0.920	0.062	M_2_ vs. M_3_	0.012	−0.002
**Major**							
M_0_ (configural)	658.715	178	0.950	0.056			
M_1_ (metric)	664.725	191	0.951	0.054	M_0_ vs.M_1_	0.001	−0.002
M_2_ (scalar)	677.409	204	0.951	0.052	M_1_ vs.M_2_	0.000	−0.002
M_3_ (strict)	687.377	219	0.951	0.050	M_2_ vs.M_3_	0.000	−0.002

MC, model comparison; ΔCFI, M_n-1_’s CFI - M_n_’s CFI; ΔRMSEA, M_n-1_’s RMSEA - M_n_’s RMSEA.

#### Criterion validity

The results showed that the correlation between the total METP Scale score and the total scores of ruminative responses (RR) and meaning in life (MIL) were 0.37 and 0.21, respectively, and the *p* values were all less than 0.001. There was a significant positive correlation between ruminative experience of time passing (RETP) and RR (*r* = 0.20, *p* < 0.001), and RR’s three dimensions (*r* = 0.13 to 0.26, *p* < 0.01). RETP was positively correlated with positive past (PPA), positive present (PPR), and positive future (PFU) (*r* = 0.15 to 0.33, *p* < 0.001). There were significant positive correlations between RETP and search for meaning (MLQ-S) and presence of meaning (MLQ-P) (*r* = 0.28 and 0.33, respectively; *p* < 0.001). There was a significant positive correlation between emotional experience of time passing (EETP) and the total RR score (*r* = 0.48, *p* < 0.001), and the score of RR’s three dimensions (*r* = 0.36 to 0.50, *p* < 0.001). There was a significant positive correlation between EETP and negative past (NPA), negative present (NPR), and negative future (NFU) (*r* = 0.31 ~ 0.40, *p* < 0.001), while EETP was negatively correlated with PPR and PFU (*r* = −0.21 and − 0.14, respectively; *p* < 0.001). Finally, there was a significant negative correlation between EETP and MLQ-P (*r* = −0.18, *p* < 0.001). The correlation coefficients among the different variables are shown in Table 6.

**Table 6:** Correlation coefficient between the two factors of METP and each dimension of the three criterion scales (*n* = 579).

	*M*	*SD*	1	2	3	4	5	6	7	8	9	10	11	12	13	14	15	16
1 RETP	37.22	5.06	1															
2 EETP	16.82	3.66	0.45^**^	1														
3 SRU	24.37	6.14	0.13^**^	0.49^**^	1													
4 BRD	11.04	2.72	0.21^**^	0.41^**^	0.81^**^	1												
5 RPD	10.34	2.52	0.26^**^	0.36^**^	0.72^**^	0.74^**^	1											
6 PPA	17.06	3.26	0.25^**^	−0.09^*^	−0.31^**^	−0.18^**^	−0.13^**^	1										
7 NPA	13.32	3.69	−0.04	0.32^**^	0.43^**^	0.28^**^	0.28^**^	−0.51^**^	1									
8 PPR	16.37	3.34	0.15^**^	−0.21^**^	−0.47^**^	−0.36^**^	−0.20^**^	0.55^**^	−0.28^**^	1								
9 NPR	14.02	3.55	0.02	0.40^**^	0.57^**^	0.45^**^	0.31^**^	−0.33^**^	0.57^**^	−0.64^**^	1							
10 PFU	17.63	3.42	0.33^**^	−0.14^**^	−0.39^**^	−0.21^**^	−0.13^**^	0.62^**^	−0.34^**^	0.70^**^	−0.44^**^	1						
11 NFU	13.37	3.09	−0.11^**^	0.31^**^	0.44^**^	0.23^**^	0.16^**^	−0.24^**^	0.56^**^	−0.34^**^	0.62^**^	−0.49^**^	1					
12 MLQ-S	24.68	5.20	0.33^**^	0.06	−0.08^*^	0.08	0.09^*^	0.31^**^	−0.08	0.27^**^	−0.08	0.38^**^	−0.16^**^	1				
13 MLQ-P	22.54	5.11	0.28^**^	−0.18^**^	−0.40^**^	−0.25^**^	−0.08	0.45^**^	−0.28^**^	0.62^**^	−0.48^**^	0.68^**^	−0.45^**^	0.49^**^	1			
14 METP	54.04	7.46	0.90^**^	0.80^**^	0.33^**^	0.35^**^	0.35^**^	0.13^**^	0.13^**^	−0.00	0.21^**^	0.16^**^	0.08	0.25^**^	0.10^*^	1		
15 RR	45.75	10.54	0.20^**^	0.48^**^	0.97^**^	0.91^**^	0.85^**^	−0.26^**^	0.39^**^	−0.42^**^	0.52^**^	−0.31^**^	0.35^**^	−0.01	−0.32^**^	0.37^**^	1	
16 MIL	47.22	8.91	0.35^**^	−0.07	−0.28^**^	−0.10^*^	0.01	0.44^**^	−0.21^**^	0.51^**^	−0.32^**^	0.61^**^	−0.35^**^	0.87^**^	0.86^**^	0.21^**^	−0.19^**^	1

M, mean; SD, standard deviation. ^*^p < 0.05, ^**^p < 0.01. RETP, ruminative experience of time passing; EETP, emotional experience of time passing; SRU, symptom rumination; BRD, brooding; RPD, reflective pondering; PPA, positive past; NPA, negative past; PPR, positive present; NPR, negative present; PFU, positive future; NFU, negative future; MLQ-S, search for meaning; MLQ-P, presence of meaning; METP, metacognitive experience of time passing; RR, ruminative responses; MIL, meaning in life.

#### Reliability analysis

The results showed that the internal consistency reliability of the total questionnaire and the RETP and EETP dimensions of the METP Scale were 0.89, 0.89, and 0.82, respectively. The split half reliability of the total Scale and the RETP and EETP dimensions of the METP Scale were 0.76, 0.88 and 0.79, respectively. The retest reliability of the total Scale and the RETP and EETP dimensions of the METP Scale were 0.78, 0.77, and 0.78, respectively.

#### Regression analysis

Using grade as a categorical variable with four levels, we set three dummy variables, taking the total METP score as the dependent variable, and gender, major, age, symptom rumination (SRU), brooding (BRD), reflective pondering (RPD), PPA, NPA, PRE, NPR, PFU, NFU, MLQ-S, and MLQ-P as the independent variables. Standard multiple regression analysis was performed for data of Sample 4. The ANOVA results of the regression equation showed that the linear regression equation was significant, *F*_(17,561)_ = 12.05, *p* < 0.001. That is, the symptom rumination (SRU), positive past (PPA), negative present (NPR), positive future (PFU), and searching for meaning (MLQ-S) were shown to significantly predict METP. The regression coefficient estimation and related results are shown in Table 7.

**Table 7:** Results of regression analysis of factors influencing METP.

Model	Unstandardized coefficients		Standardized coefficients	*t*	Sig.	Collinearity statistics	
	β	SE	β			Tolerance	VIF
(Constant)	22.75	5.95		3.83	0.00		
Major	−0.35	0.58	−0.02	−0.60	0.55	0.85	1.17
Grade 1	0.27	0.66	0.02	0.41	0.68	0.67	1.49
Grade 2	2.35	1.33	0.07	1.77	0.08	0.74	1.34
Grade 3	1.19	1.68	0.03	0.71	0.48	0.58	1.73
Age	−0.21	0.26	−0.04	−0.81	0.42	0.58	1.71
Gender	−0.10	0.64	−0.1	−0.16	0.87	0.97	1.04
SRU	0.34	0.10	**0.28**	3.51	**0.00**	0.21	4.84
BRD	0.09	0.20	0.03	0.46	0.65	0.26	3.91
RPD	0.31	0.18	0.10	1.73	0.08	0.36	2.79
PPA	0.27	0.13	**0.12**	2.12	**0.03**	0.41	2.43
NPA	0.09	0.12	0.04	0.77	0.44	0.41	2.47
NPR	0.29	0.14	**0.14**	2.06	**0.04**	0.29	3.41
PFU	0.46	0.15	**0.21**	3.17	**0.00**	0.30	2.56
NFU	−0.03	0.14	−0.01	−0.20	0.85	0.39	2.56
NPR	−0.08	0.15	−0.04	−0.55	0.58	0.28	3.61
MLQ-S	0.21	0.06	**0.15**	3.31	**0.00**	0.67	1.48
MLQ-P	0.08	0.09	0.05	0.92	0.36	0.39	2.59

SRU, symptom rumination; BRD, brooding; RPD, reflective pondering; PPA, positive past; NPA, negative past; PPR, positive present; NPR, negative present; PFU, positive future; NFU, negative future; MLQ-S, search for meaning; MLQ-P, presence of meaning. VIF, variance inflation factor; SE, standard error; Sig, significance.

## Discussion

Based on the S-Shape Model proposed by Flaherty (2018), the concept of metacognition proposed by Flavell (1976, 1979), and the thought of time passing in Chinese time culture, this study explores the connotation and structure of Chinese college students’ experience of time passing (ETP). All three backgrounds are reflected in the results of this study to a certain extent: First, the S-Shape Model lays great emphasis on the influence of experiential density (ED) on the experience of time passing, and ED mainly depends on cognitive involvement. In fact, the present study did confirm the dimension of thinking experience in the self-developed scale, and the content of this dimension is consistent with the cognitive complexity and significance proposed by Flaherty (2018). Second, the two dimensions of metacognitive experience of time passing (METP), ruminative experience of time passing (RETP) and emotional experience of time passing (EETP), were seen to have distinct scores separate from the overall METP Scale, which is in line with concept of metacognitive experience raised by Flavell, which proposes that experience includes both a cognitive and emotional component (Wang and Guo, 2000). Third, Chinese traditional time culture emphasizes the harmony and unity of time, space, people and events. From the perspective of logical reasoning, Chinese people’s ETP is also likely to be affected by the abovementioned factors. Interestingly, this inference is also reflected in the items of the self-developed scale, such as, “The sense of time passing reminds me that I need to think about changing things in my life” “The sense of time passing makes me think about my relationship to my surroundings” “The sense of time passing reminds me to do things” “The sense of time passing makes me think about my relationships with others,” etc.

Next, we will discuss the structural stability and credibility of the scale developed in this study from METP, RETP and EETP, respectively. First, the METP Scale was shown to meet various measurement standards, and showed measurement invariance in gender, grade, and major, indicating that it has a relatively stable structure and is an ideal standardized tool to use to evaluate METP of college students. Second, Compared to the other dimensions of the Rumination Scale (RS), reflective pondering (RPD) had the highest correlation with the RETP dimension in the METP Scale (*r* = 0.26). The reason for this could be that the METP mainly focuses on the past (Tipples, 2018), and involves a richness of ideas and advanced thinking processes such as reasoning and memory (Yu et al., 2018a). The content of the RETP dimension in the METP Scale can be summarized as time-self, growth, and interpersonal relationship reflection, which is in alignment with the integrated concept of time–space-people-events in Chinese traditional time culture (Su and Ye, 2020). Rumination, meanwhile, focuses mainly on negative experiences or stressful events, and findings have suggested that rumination about past failures is related to time awareness (Tipples, 2018). Additionally, there was a significant positive correlation between the RETP dimension scores and the positive past, positive present, and positive future constructs of the Time Attitude Scale, which also reflects the positive significance of experience of time (Yu et al., 2017). Furthermore, there is a significant positive correlation between RETP and both MLQ-S and MLQ-P, which further confirms that the experience of time is not only the experience of time itself, but also includes self-concept, self-worth, and sense of meaning (Twenge et al., 2003; Droit-Volet and Dambrun, 2019), which can be referred to as the philosophical horizon of time and being. With regards to the EETP dimension of the METP Scale, this dimension ultimately included not only all the items of the emotional dimension of the Feeling of the Passage of Time Scale (Yu et al., 2017), but also increased by one item, indicating the richness of EETP. EETP in this study was still dominated by negative introspective emotion, which may be related to the tendency of individuals to construct a negative sense of self (Zhang et al., 2021). It is worth noting that the correlation between EETP and each dimension of the Rumination Scale was higher than that between RETP and the same dimensions of the Rumination Scale. As rumination is most often repeated thought patterns in response to stressful events (Han and Yang, 2009), it leads to more negative experiences; meanwhile, EETP is also a type of negative introspective emotion (see items in Table 3 for details). These results are consistent with the previous conclusion in that ruminative thinking is more likely to induce negative emotion (Jahanitabesh et al., 2019). In addition, RETP was found to have a significant positive correlation with PPA, PPR, and PFU, no relationship with NPA and NPR, but a significant negative correlation with NFU (*r* = −0.11, *p* < 0.01). Meanwhile, EETP showed a significant negative correlation with PPA, PPR, and PFU, and a positive correlation with NPA, NPR, and NFU. These inconsistencies between thinking and emotional experience from a time perspective (TP) could be because, from the perspective of the function of ETP, RETP in fact focuses on the future, as the present and future states can be changed, but the past cannot be changed. Therefore, RETP has a positive significance. The results also showed that RETP is significantly positively correlated with MLQ-S and MLQ-P, with a higher correlation to MLQ-S. However, EETP has negative correlations to both. In addition to a significant negative correlation with positive time attitude and a positive correlation with negative time attitude, EETP also has a significant negative correlation with MLQ-P. The past is neither reversible nor changeable, and when looking to the past in the context of the passage of time, one is more likely to experience a sense of frustration or absence.

Multiple regression analysis showed that symptom rumination (SRU), positive past (PPA), negative present (NPR), positive future (PFU), and search for meaning (MLQ-S) could significantly predict the intensity of METP. Placed in the order of their standardized regression coefficient, the variables affecting the intensity of METP were found to be SRU, PFU, MLQ-S, NPR, and PPA.

First, SRU is based on depressive symptoms (Alonso et al., 2004; Mello and Worrell, 2007; Han and Yang, 2009), and previous studies have shown that perception of time passing slowly is one of the common symptoms of depression, and is also particularly related to the severity of intellectual disability and emotional disorders (Blewett, 1992). Therefore, it is not surprising that SRU can predict METP. SRU, as the primary predictor among the selected variables, may indicate that METP is dominated by the experience of thinking. Meanwhile, the number of items in the RETP dimension (i.e., twice as many as the EETP dimension) further points to the fact that thought has a strong influence on METP overall.

Second, the results of regression analysis showed that several dimensions of time attitude, positive future (PFU), negative present (NPR), and positive past (PPA), can significantly predict METP. Previous studies have shown that negative past, fatalistic present, and future time perspective in the Zimbardo Time Perspective Inventory significantly predicted the feeling of the passage of time (Yu et al., 2018a,b). Comparing the current study with that of Yu et al. (2018b), with the exception of the same predictive variable of negative attitude towards the present, there was completely opposite valence in all other predictive variables of the time perspective. For example, Yu et al. (2018b) found that negative past (NPA) significantly predicts the feeling of the passage of time (FPT), while in the present study, it is the positive past (PPA) that significantly predicts METP. The reason for this difference could be that the two studies differ greatly in terms of their measurement constructs. Yu et al. (2018b) measured the feeling of the passage of time, in which the negative emotion of time passing and susceptibility occupy the construct. In the current study, however, RETP comprises the dominant part of the scale. As mentioned above, the RETP items have a tendency towards the positive.

Third, the results of regression analysis also showed that search for meaning (MLQ-S), rather than presence of meaning (MLQ-P), significantly predicted METP. It should be noted that MLQ-P refers to the degree to which an individual feels that their life is meaningful (i.e., emphasizing cognitive results), while MLQ-S refers to the degree to which an individual actively searches for meaning, i.e., emphasizing cognitive processes (Steger et al., 2006; Wang, 2013). Given that individuals generally tend to underestimate their self-worth and construct a more negative sense of self (Zhang et al., 2021), individuals’ responses to the question, “Do you feel you have meaning in your life?,” may have been more conservative and negative. Furthermore, the scale developed in this study places emphasis on cognitive experience, which is consistent with the characteristics of searching for meaning in life emphasizing the cognitive process. In addition, it takes time for one to figure out their meaning of life. Therefore, the conclusion that MLQ-S can predict the intensity of METP is credible.

## Conclusion

The METP is the consciousness of the ongoing march of life. Strengthening one’s METP can be helpful in building life management skills, especially for college students who are still in a state of strong plasticity. Through literature analysis, open and semi-structured interviews, and incorporation of the concepts of traditional Chinese time culture, this study developed and verified the METP Scale testing experience of time passing. Through item analysis and EFA, 15 items were extracted, which comprised the metacognitive ruminant experience as well as the emotional experience of time passing (RETP & EETP). The reliability and validity test of the METP Scale was shown to meet psychometrics standards, and our findings show that it can be used as a tool to test METP in Chinese college students.

## Limitations and prospects

Experience of time passing has been shown to be influenced by gender, age, socioeconomic status, and situational state (Yu et al., 2018a, 2022). Although the structure of the METP Scale developed in the current study has measurement invariance in gender, grade, and major, there are still a large number of differences in the demographic variables of our samples. Therefore, future studies need to balance the distribution of the number of participants such that the impact of other demographic variables can be assessed. At the same time, potential profile analysis could be considered to detect whether there are different idiosyncrasies present in the METP Scale. Furthermore, cross-cultural research is needed to explore and verify the structure of the METP Scale in other geographic or cultural regions. Finally, scholars have long believed that hardly anyone questions the importance of metacognition, which is crucial for cognitive monitoring, emotional regulation, and strategy choice (Schraw and Moshman, 1995; Moshman, 2018). Similarly, metacognitive experience of time passing (METP) will also affect college student’s cognition, emotion, and thinking of time. Therefore, a structural equation model should be constructed by combining the METP Scale with other variables, such as college students’ learning engagement, achievement motivation, subjective well-being, and meaning in life.

## Data availability statement

The original contributions presented in the study are included in the article/Supplementary material, further inquiries can be directed to the corresponding author/s.

## Ethics statement

The studies involving human participants were reviewed and approved by Ethics Review Committee of School of Education Science, Guangdong Polytechnic Normal University. Written informed consent to participate in this study was provided by the participants’ legal guardian/next of kin.

## Author contributions

XY and JL participated in the research design, interview organization, questionnaire preparation and testing, data collation and analysis, and manuscript writing and proofreading. YL participated in the writing, proofreading, and revision of the manuscript. XC and CL participated in the recruitment of participants and questionnaire testing. All authors contributed to the article and approved the submitted version.

## Funding

This study received funding from the Guangdong Province Philosophy and Social Sciences “13th Five-Year Plan” project: Metacognitive Research on the feeling of the passage of time (Project NO. GD20XXL05).

## Acknowledgments

We would like to thank all the college students who participated in the study and the teachers who organized the testing. We would also like to thank the editors, reviewers, and other members of the editorial office for their help with this article. Furthermore, we would like to thank Professor Michael G. Flaherty for his generosity.

## Conflict of interest

The authors declare that the research was conducted in the absence of any commercial or financial relationships that could be construed as a potential conflict of interest.

## Publisher’s note

All claims expressed in this article are solely those of the authors and do not necessarily represent those of their affiliated organizations, or those of the publisher, the editors and the reviewers. Any product that may be evaluated in this article, or claim that may be made by its manufacturer, is not guaranteed or endorsed by the publisher.
