# Supplementary material for: The metacognitive experience of time passing in Chinese college students: scale development, structure verification, and influencing factors
Source: Front Psychol. 2023 Jun 28;14:1180863. doi: 10.3389/fpsyg.2023.1180863 (PMC10338092; doi:10.3389/fpsyg.2023.1180863)
Supplement: Supplementary file 1 [file Table_1.docx]

**Supplementary Material**

**Item analysis of sample 3**

Using data of sample 3, the results of reliability analysis showed that the internal consistency reliability of the METP Scale was 0.889. Referring to the item analysis section of the main text, we tested to see whether the Cronbach’s *α* coefficient of the scale would be significantly improved after the deletion of each individual item. The results showed that the reliability of the questionnaire remained between 0.880 and 0.885 after each deletion of an item, which was lower than the initial reliability of 0.889 when all items were included. See Table 8 for details.

Table 8

***Results of item-total statistics (sample 3, n = 1712)***

| Item  number | Scale average if item deleting | Scale variance if item deleting | Corrected item-total correlation | Alpha if item deleted |
| --- | --- | --- | --- | --- |
| N1 | 48.67 | 45.253 | 0.568 | 0.881 |
| N2 | 48.85 | 44.787 | 0.601 | 0.880 |
| N3 | 48.73 | 44.665 | 0.627 | 0.879 |
| N4 | 48.81 | 45.065 | 0.597 | 0.880 |
| N5 | 48.66 | 45.683 | 0.534 | 0.882 |
| N6 | 48.83 | 44.710 | 0.606 | 0.879 |
| N7 | 48.97 | 45.605 | 0.502 | 0.884 |
| N8 | 48.87 | 45.088 | 0.574 | 0.881 |
| N9 | 48.72 | 45.426 | 0.539 | 0.882 |
| N10 | 48.69 | 44.703 | 0.600 | 0.880 |
| N11 | 49.13 | 44.473 | 0.531 | 0.883 |
| N12 | 49.34 | 45.148 | 0.470 | 0.885 |
| N13 | 49.13 | 44.299 | 0.539 | 0.882 |
| N14 | 49.16 | 44.479 | 0.556 | 0.881 |
| N15 | 49.22 | 45.002 | 0.502 | 0.884 |

**Item analysis of sample 4**

Using data of sample 4, the results of reliability analysis showed that the internal consistency reliability of the METP Scale was 0.892. Referring to the item analysis section of the main text, we tested to see whether the Cronbach’s *α* coefficient of the scale would be significantly improved after the deletion of each individual item. The results showed that the reliability of the questionnaire remained between 0.883 and 0.890 after each deletion of an item, which was lower than the initial reliability of 0.892 when all items were included. See Table 9 for details.

Table 9

***Results of item-total statistics (sample 4, n = 579)***

| Item  number | Scale average if item deleting | Scale variance if item deleting | Corrected item-total correlation | Alpha if item deleted |
| --- | --- | --- | --- | --- |
| N1 | 50.31 | 48.579 | 0.621 | 0.883 |
| N2 | 50.26 | 49.275 | 0.610 | 0.884 |
| N3 | 50.13 | 50.438 | 0.446 | 0.890 |
| N4 | 50.61 | 49.252 | 0.500 | 0.888 |
| N5 | 50.42 | 49.181 | 0.574 | 0.885 |
| N6 | 50.02 | 50.344 | 0.530 | 0.887 |
| N7 | 50.27 | 49.516 | 0.560 | 0.886 |
| N8 | 50.53 | 48.890 | 0.554 | 0.886 |
| N9 | 50.41 | 48.406 | 0.621 | 0.883 |
| N10 | 50.20 | 49.528 | 0.583 | 0.885 |
| N11 | 50.34 | 47.854 | 0.602 | 0.884 |
| N12 | 50.79 | 48.274 | 0.512 | 0.888 |
| N13 | 50.72 | 47.011 | 0.621 | 0.883 |
| N14 | 50.62 | 47.422 | 0.609 | 0.883 |
| N15 | 50.90 | 48.048 | 0.534 | 0.887 |

**Reliability analysis of sample 2**

The results showed that the internal consistency reliability of the total questionnaire and the RETP and EETP dimensions of the METP Scale were 0.863, 0.852, and 0.819, respectively. The split-half reliability of the total Scale and the RETP and EETP dimensions of the METP Scale were 0.702, 0.798, and 0.828, respectively.
